# Exploring public attitudes of continuing care retirement communities in China: a sentiment analysis of China’s social media Weibo

**DOI:** 10.3389/fpubh.2024.1454287

**Published:** 2025-02-18

**Authors:** Xuechun Wang, Bo Xia, Qing Chen, Martin Skitmore, Huiming Liu

**Affiliations:** ^1^Department of Engineering Management, Shanxi Vocational University of Engineering Science and Technology, Taiyuan, Shanxi Provience, China; ^2^School of Architecture and Built Environment, Queensland University of Technology, Brisbane, QLD, Australia; ^3^University Professorial Fellow, Faculty of Society and Design, Bond University, Robina, QLD, Australia; ^4^Faculty of Humanities and Art, Macau University of Science and Technology, Taipa, Macau, China

**Keywords:** aging, continuing care retirement communities, natural language processing, public attitudes, social media, sentiment analysis, Weibo

## Abstract

**Introduction:**

The traditional family responsibility system faces challenges as China undergoes rapid demographic shifts with an increasingly older population. Recognizing the potential of market-driven senior care, Continuing Care Retirement Communities (CCRCs) have emerged as a significant alternative. However, cultural stigmas and concerns about the quality, services, and health of older adults in these facilities raise questions over their broad acceptance.

**Methods:**

This study examines public sentiment toward CCRCs through sentiment analysis of 1,027,295 pre-processed Weibo posts. Utilizing Natural Language Processing (NLP) combined with fine-grained sentiment analysis and the Term Frequency-Inverse Document Frequency algorithm, the attitudes and emotions reflected in each data point are analyzed, identifying key contributing factors, and exploring the underlying reasons.

**Results and discussion:**

The results reveal a predominantly positive sentiment toward CCRCs, emphasizing factors such as the living environment and government involvement. However, areas of concern, such as potential fraud and health and safety issues, remain. These findings both shed light on the public’s acceptance or resistance to CCRCs for stakeholders and highlight the potential of social media analysis in shaping older people’s care perceptions in today’s China.

## 1 Introduction

Rapid aging is a pressing concern globally. According to the projections from the United Nations (1), the global proportion of older individuals will rise to 12% (994 million) in 2030, followed by a further increase to 16% (1.6 billion) in 2050, doubling the current senior population of 771 million. This increase is occurring at an unprecedented pace and will accelerate in the coming decades, particularly in developing countries. As the world’s most populous developing nation, China is facing unprecedented growth in its older population (61). It has been an “aging society” since 2000 (2) and has already replaced Japan as the quickest-aging country in the world. The National Working Committee on Aging (3) forecasted that the proportion of older Chinese will rise to 18.2% by 2030, which is expected to grow to 27.9% (approximately 400 million) by 2050.

In the past, China benefited from a fairly robust system of family responsibility through which older adults could rely on substantial assistance (74). However, the diminution of family structures and other societal transformations have increased the strain on senior care, therefore upending conventional home-based care for older people (4). These tendencies will be a significant social and public issue, hindering China’s long-term sustainable growth. In response, the Chinese authorities have progressively understood that market-driven senior care might effectively address the problem of limited family-based older adult care capacities (5) and made it explicit that comprehensive support for commercialized senior care institutions would be reinforced.

Against this background, continuing care retirement communities (CCRCs) are emerging in China as an alternative residential arrangement for the aging population predominantly funded by the market (67). It provides a diverse range of housing and facility selections with evolved life caring and health services pertaining to the aging process for senior citizens (6). The emergence of CCRCs can be attributed to their appealing age-friendly built environments, the extensive range of specialized services tailored to senior citizens, such as emergency relief and priority access in collaboration with local top-tier hospitals, as well as the supportive and engaging social environment (7, 73).

Despite the potential benefits of CCRCs in addressing the housing and service needs of China’s aging population, a cultural stigma still exists (59). However, to a lesser degree, surrounding institutionalized care for older people due to traditional values of filial piety, i.e., it is regarded as immoral and unfilial to hand over older parents to institutions to spend their later years in China as conventional home-based senior care is frequently linked to the local value of “filial piety” (8, 71). Additionally, concerns from older people and the public over the quality of facilities, services, and environment within CCRCs have been raised, given the relative novelty of this type of care in China. These concerns from the public may hinder the adoption of CCRCs as a viable long-term care solution for China’s aging population.

Therefore, it is important to have a holistic understanding of the general public’s attitudes/sentiments toward CCRCs in China. This knowledge is valuable for informing policymakers, investors, and developers concerning CCRCs’ viability and acceptance in the Chinese context. Such understanding shall also aid policymakers in crafting policies that promote and regulate CCRCs development and protect consumer interests while helping investors make informed decisions in this rapidly expanding market. Furthermore, public feedback enables CCRCs operators to refine services, facilities, and care models, ensuring alignment with residents’ and families’ expectations and facilitating ongoing quality enhancement by gauging public sentiment.

Despite its significant importance, the general public’s attitude toward CCRCs in China has not been adequately examined due to the relatively limited public awareness of CCRCs in past years and, more importantly, the demanding nature of data sources and the required sample size to assess public sentiment. Fortunately, social media analysis has emerged in recent years as a pivotal tool for understanding societal trends and behaviors. Social media platforms are gaining popularity in the new era of big data, offering distinct benefits over traditional media channels, such as real-time data collection on an unprecedented scale and capturing people’s awareness and response to emerging and major trending topics (9). Due to these characteristics, social media is an alluring data source and laboratory for social science research. Like Twitter and Facebook, Weibo, the most influential localized social media network in China, is becoming acknowledged as a robust database in applications that deliver diverse and authentic viewpoints and potential sentiment evaluations.

Therefore, a comprehensive sentiment analysis of Weibo posts in China was conducted to understand the general public’s perception and attitude toward CCRCs in China. More specifically, this study concentrates on examining three progressive objectives: (1) classifying Weibo users’ sentiment regarding CCRCs based on Ekman’s theory of emotions (positive, neutral, negative); (2) categorizing emotions (also known as fine-grained sentiment analysis) into seven categories—“happiness,” “good,” “anger,” “sadness,” “fear,” “disgust,” and “surprise”—and 21 sentimental sub-categories based on the Dalian University of Technology Chinese Sentiment Vocabulary Classification System derived from Ekman’s Theory; and (3) utilizing the Term Frequency-Inverse Document Frequency (TF-IDF) algorithm to extract the key sentimental vocabulary of each sub-category.

## 2 Methods

Sentiment analysis, or opinion mining, employs computational and linguistic techniques to identify and extract subjective information (10). Its primary strength lies in its ability to extract the emotional tone from extensive volumes of text. In the contemporary digital era, public sentiment captured through social media platforms provides valuable insights into stakeholder perspectives. Selecting an authoritative, widely recognized, and accepted platform is critical for ensuring unbiased insights. In the Chinese digital landscape, Weibo, similar to its global counterpart Twitter, is known for its extensive user base and role in stimulating discussions on current issues. A Sentiment Analysis Framework is followed based on earlier research (11), integrating traditional linguistic algorithms with modern machine-learning techniques to ensure a comprehensive and replicable approach.

### 2.1 Sentiment analysis of the general public toward CCRCs

Sentiment refers to an individual’s attitude and subjective experience regarding the extent to which objective entities meet their needs. It encompasses expressions such as liking and disgust (76). The emotional response to CCRCs—whether positive or negative—can provide stakeholders with critical feedback on public acceptance or resistance to such living arrangements.

Scholarly perspectives on the dimensional categorization of sentiment vary. Wundt’s sentiment model includes pleasantness-unpleasantness, tension-relaxation, and excitement-inhibition (12). Titchener (13) argued that bodily sensations are integral to tension-relaxation and excitement-inhibition, contending instead that sentiment can be characterized by a single dimension: pleasantness-unpleasantness. Traditional Chinese sentimental thought features various categorizations, such as the 7, 6, 5, and 3 sentiments. Wang et al. (14) further distinguished sentiment into two dimensions: positive and negative. According to Fredrickson’s (15) broaden-and-build theory, positive sentiment is more advantageous than negative sentiment in expanding attention, cognition, and behavior. Current consensus indicates that emotion significantly influences behavior (16).

In the context of CCRCs, the sentiment expressed on Weibo gauges the general public’s acceptance and concerns, offering insights into the sector’s potential growth. Positive sentiment toward CCRCs on Weibo may indicate broader acceptance and could lead to increased exploration and adoption of such communities. Conversely, negative sentiment might impede or even reduce the future uptake of CCRCs.

### 2.2 Leveraging Weibo for sentiment analysis of CCRCs

Public acceptance, presentation, and dissemination of information collectively shape the public’s perception of social issues and emerging trends. Traditionally, people have relied on experts, government, and media as authoritative sources of information (17). However, social media has increasingly become a prominent tool for social interaction (18), offering insights into not only the development of CCRCs but also the emotional responses of the public.

In recent years, Weibo, a Chinese microblogging platform, has experienced rapid growth as a social network. An increasing number of individuals express their opinions and emotions through Weibo. According to Sina Finance (19), Weibo had over 586 million monthly active users and up to 252 million daily active users by the end of 2022, with the user base growing. The platform has become a hub for discussing popular events and themes, generating vast data rich in subjective emotional content reflecting everyday experiences. Efficiently identifying and analyzing these emotional trends is valuable for understanding public sentiment. Sentiment analysis involves mining and analyzing user-generated information and helps identify sentiment trends. By utilizing sentiment classification technology, it is possible to discern the emotional tendencies of Weibo users. This analysis aids developers and policymakers in understanding user needs in the care sector, thereby enhancing the efficiency and effectiveness of CCRC services.

### 2.3 Sentiment analysis framework and process for public sentiment of CCRCs on Weibo

**Figure 1:**
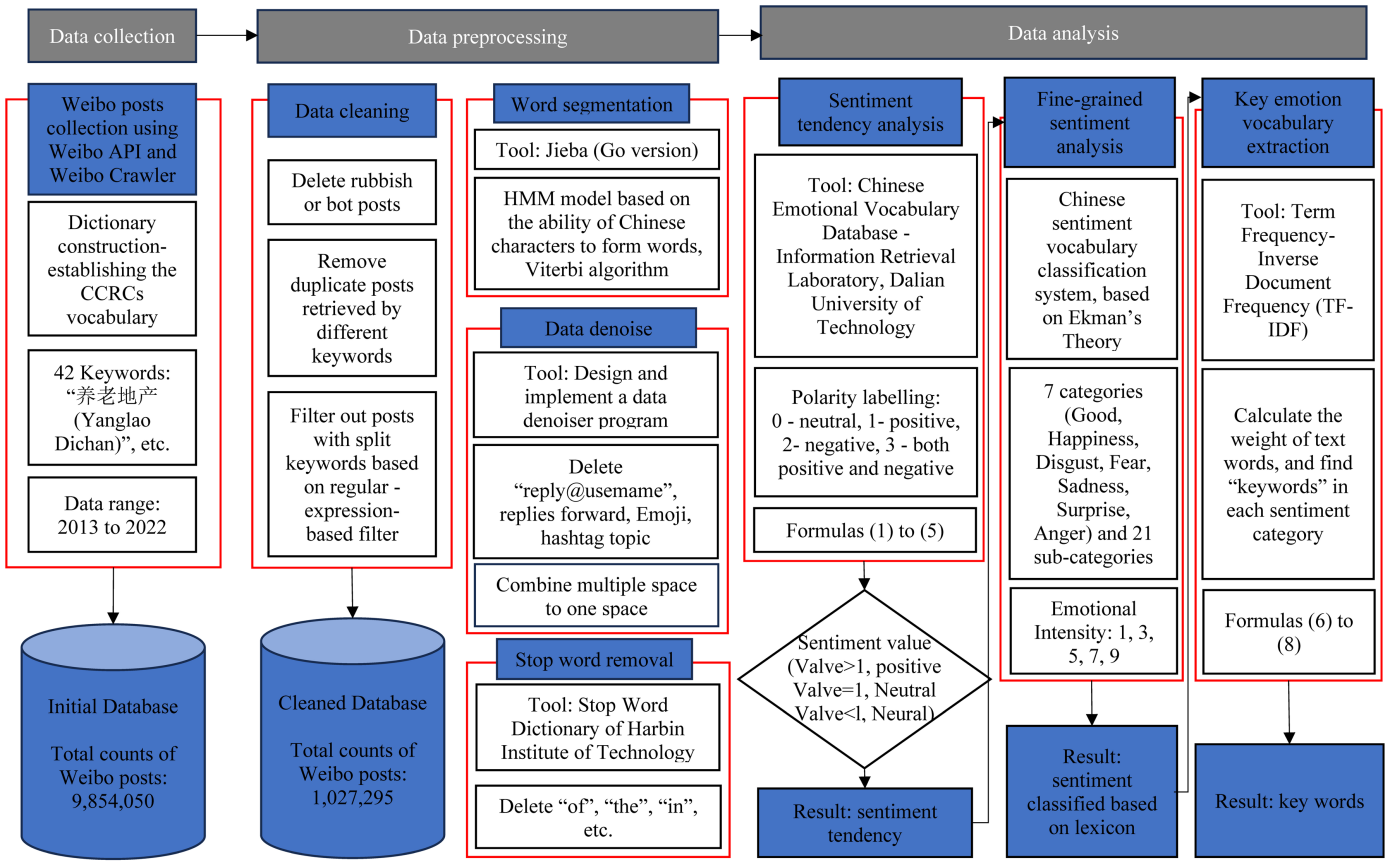
The flow chart of the sentiment analysis.

The strategy illustrated in Figure 1 was employed to investigate the general public’s perception of CCRCs in China. The study was divided into three main phases. The first phase involved establishing a vocabulary for CCRCs, which served as a set of defined keywords for searching and browsing Weibo (often called “Chinese Twitter”). The second phase encompassed data processing, where collected posts relating to CCRCs were cleaned, segmented, denoised, and stripped of stop words to retain only emotionally relevant terms for subsequent analysis. The final phase focused on data analysis, assessing the emotional dispositions of individuals toward CCRCs. In this phase, initially, the analysis, grounded in the affective tendency theory from the Dalian University of Technology (20), categorized the emotional inclinations of CCRC posts as positive, negative, neutral, or a combination of positive and negative. Following this, a fine-grained classification approach was used to detail individual sentiment classifications from Weibo reviewers further. The concluding step involved extracting key sentiment words and calculating their weight using the TF-IDF algorithm.

### 2.4 Phase 1: data collection

#### 2.4.1 Step 1 dictionary construction

**Figure 2:**
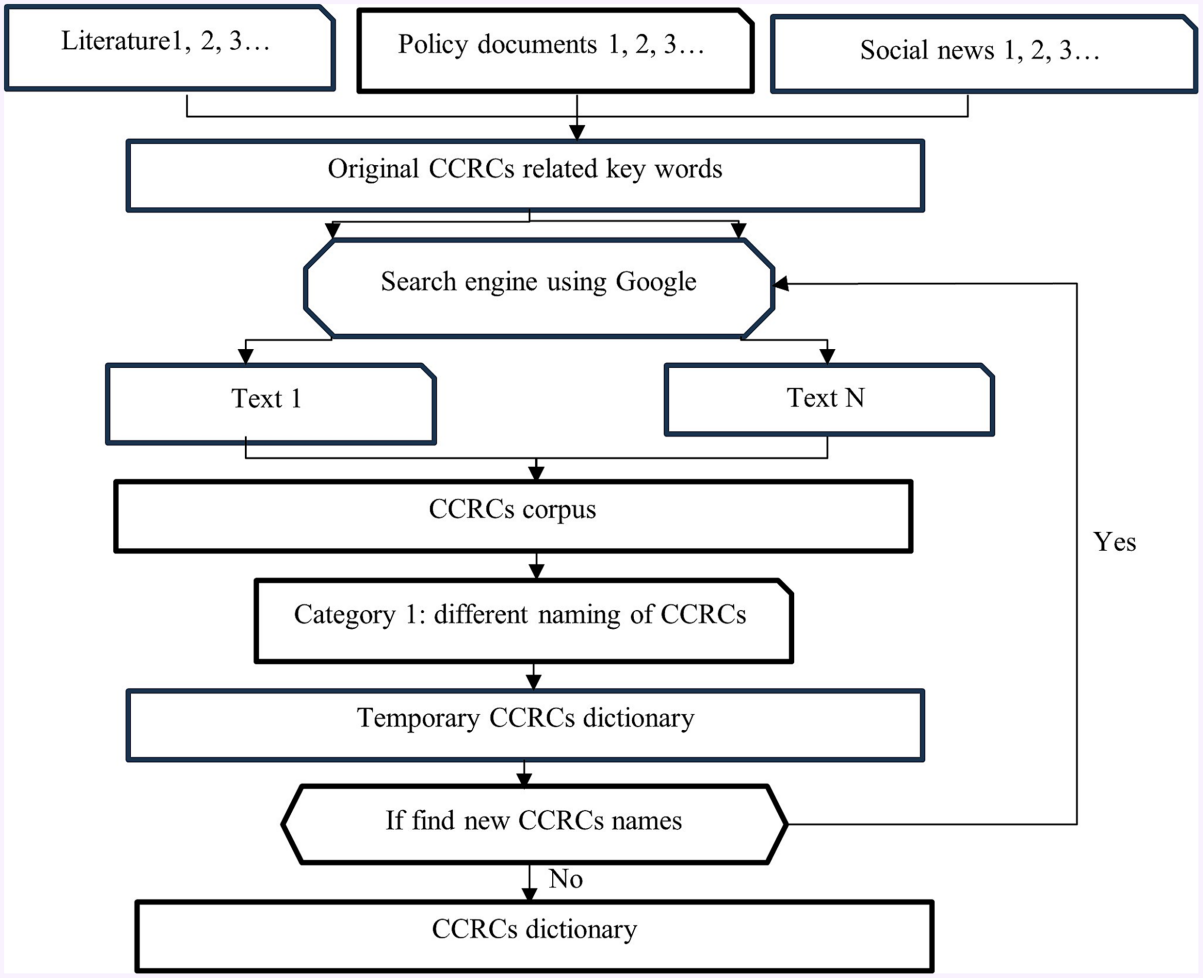
Dictionary construction.

To construct the dictionary, texts related to CCRCs—including academic papers, policy documents, and news articles—were compiled to gather alternative names and original keywords for CCRCs (Figure 2). The initial step involved conducting independent searches for these original keywords and retrieving the top 100 search results using Google, which was chosen for its prominence and influence (6). The resulting documents formed the corpus of CCRC-related data, integrating both search engine results and contextual information. Following a careful manual identification process, only the most relevant terms associated with CCRCs were selected for inclusion in a “temporary CCRCs dictionary” alongside the initial keywords. This iterative process was repeated until no new keywords were identified, signaling the completion of the final “CCRCs dictionary.” Convergence was achieved after five iterations, resulting in the incorporation of 500 relevant texts. The finalized CCRC dictionary is detailed in Appendix 1.

#### 2.4.2 Step 2 data collection

The research data consisted of Weibo posts obtained via the Weibo application programming interface (API) and supplemented by a Python-based web crawler after reaching the API’s data retrieval limit of 2,000 comments. The data collection methodology is detailed in Table 1. Given that Weibo’s search function includes filters not available to non-users, the crawler program retrieved publicly accessible data by emulating a regular end user. This process involved creating a Weibo account, with the crawler program utilizing the account’s authorization information to simulate typical user access. The data collection period ranged from January 1, 2013, considered the inaugural year for CCRCs, to November 28, 2022.

**Table 1:** Weibo data-fetching method.

Step 1: Weibo API
Establish a Weibo application and acquire an APP Key
Request authentication and API interface access
Parse returned JSON data
Save the retrieved textual information in CSV format
Step 2: Weibo web crawler
Create a Weibo account
Sign into the Weibo account via the browser’s built-in tools for developers and obtain session cookie
Set cookie and other search parameter in crawler program
Imitate a typical Weibo user and instruct the crawler to visit websites that correspond to a definite search parameter
Using regular expression, the crawler program pulls pertinent data from web pages
Save the extracted data in the CSV format

### 2.5 Phase 2: data cleaning and pre-processing

Following data acquisition, data cleansing was the initial step before pre-processing. Weibo posts with fragmented keywords required cleaning, as the search engine sometimes segmented keywords, resulting in incomplete keyword representation in the collected posts. Additionally, duplicate posts needed to be removed because Weibo posts were retrieved multiple times due to the simultaneous crawling of 41 keywords with similar meanings. Furthermore, similar to other social media platforms, Weibo search results may include irrelevant or automated content that could disrupt semantic analysis. A regular expression-based filter was used to identify and remove invalid posts. The pseudo-code for this procedure is presented in Appendix 2. After cleansing, 1,027,295 Weibo posts suitable for subsequent data analysis were identified.

Following data cleaning, three text pre-processing algorithms were developed using the Go programming language to prepare the data for sentiment analysis. The first pre-processing step involved text segmentation, which is necessary due to the continuous nature of Chinese text, where spaces do not separate words as in English. The Chinese Text Segmentation Toolkit (Jieba), implemented in Python, was utilized for this purpose. For a detailed explanation of Jieba’s functionality, refer to https://github.com/fxsjy/jieba. The second pre-processing step was denoising, which involves removing extraneous elements from Weibo comments such as “reply@username:,” “forward Weibo post,” “#Weibo topic#,” and emojis, as these can compromise the analysis’s accuracy. The final step was the removal of stop words, which are functional words without substantive meaning, such as “of,” “in,” and “the.” The Chinese Stop Word Dictionary from the Harbin Institute of Technology was used to eliminate stop words, with each segmented word checked against this dictionary to remove exact matches. An example of the entire data pre-processing procedure is provided in Appendix 3.

### 2.6 Phase 3: data analysis

Text mining, a statistical analysis tool, has been extensively applied in various fields, including biomedical analysis, online public opinion research, and market studies. Sentiment analysis, a critical method within text mining, is widely used to explore opinions on such microblog platforms as Twitter. Using Natural Language Processing (NLP) and text-analytic techniques, sentiment analysis evaluates people’s attitudes, emotions, dispositions, and moods (21). For example, user feedback on a product can be a key indicator for its improvement (10). Sentiment analysis is typically divided into three levels—vocabulary, sentence, and textual—based on the structure of the text. Given the 140-word limit of Weibo comments, which results in brief phrases and sentences, lexical and sentence analysis were deemed appropriate.

The overall sentiment tendencies of Weibo posts were initially investigated. However, such analysis was coarse-grained, as it only assessed the general sentiment trend (22). Coarse-grained sentiment analysis lacks detail regarding the specific emotional categories of individual reviewers and does not provide actionable insights for decision-making. To address this limitation, a fine-grained sentiment analysis approach was employed based on the sentiment lexicon developed by the Dalian University of Technology (77). This approach identified and categorized sentiment surrounding CCRCs within Weibo texts by extracting fine-grained emotional words and calculating their frequency and significance using the TF-IDF algorithm. The most critical emotional vocabularies were thus determined. The sentiment analysis process is illustrated in Figure 1.

#### 2.6.1 Step 1 sentiment tendency analysis

The 2021 edition of the authorized Simplified Chinese Affective Lexicon Ontology was utilized. This lexicon, developed and published by the Information Retrieval Research Laboratory at Dalian University of Technology (77), is a comprehensive collection of emotional vocabulary relevant to social networks in mainland China. The database contains 27,466 emotional words in Excel format, with annotations specifying each word’s polarity, part of speech, and emotional intensity (see the Affective Lexicon Ontology documentation). The polarity is categorized as follows: 0 indicates neutrality, 1 indicates positivity, 2 indicates negativity, and 3 indicates both positivity and negativity. The lexicon includes seven part-of-speech tagging categories: nouns, verbs, adjectives, adverbs, online words, idioms, and prepositional phrases. Emotional intensity is graded on a scale from 1 to 9, where level 1 represents the weakest intensity and level 9 represents the strongest.

The sentiment trend of Weibo posts related to CCRCs was analyzed based on the emotional words identified in this lexicon. The calculation formulas are detailed in Equations 1–5.


(1) $$\begin{array}{l} \mathrm{SentimentWordCount}=\\ \;\overset{n}{\underset{i}{\sum }}v\left(i\right)\{\begin{array}{l} v\left(i\right)=1,\mathrm{if}\;\mathrm{word}\;i\;\mathrm{is}\;\mathrm{included}\;\mathrm{in}\;\mathrm{dictionary}\\ v\left(i\right)=o,\mathrm{if}\;\mathrm{word}\;i\;\mathrm{is}\;\mathrm{excluded}\;\mathrm{from}\;\mathrm{dictionary} \end{array} \end{array}$$

(2) $$\begin{array}{l} \mathrm{SentimentPositivePct}=\\ \;\frac{\sum_{i}^{n}v\left(i\right)\{\begin{array}{c} v\left(i\right)=1,\mathrm{if}\;\mathrm{assigned}\;\mathrm{value}\;\mathrm{of}\;\mathrm{word}\;i=1\\ v\left(i\right)=0,\mathrm{if}\;\mathrm{assigned}\;\mathrm{value}\;\mathrm{of}\;\mathrm{word}\;i\neq 1 \end{array}}{\mathrm{SentimentWordCount}} \end{array}$$

(3) $$\begin{array}{l} \mathrm{SentimentNegativePct}=\\ \;\frac{\sum_{i}^{n}v\left(i\right)\{\begin{array}{c} v\left(i\right)=1,\mathrm{if}\;\mathrm{assigned}\;\mathrm{value}\;\mathrm{of}\;\mathrm{word}\;i=2\\ v\left(i\right)=0,\mathrm{if}\;\mathrm{assigned}\;\mathrm{value}\;\mathrm{of}\;\mathrm{word}\;i\neq 2 \end{array}}{\mathrm{SentimentWordCount}} \end{array}$$

(4) $$\begin{array}{l} \mathrm{SentimentNeutralPct}=\\ \;\frac{\sum_{i}^{n}v\left(i\right)\{\begin{array}{c} v\left(i\right)=1,\mathrm{if}\;\mathrm{assigned}\;\mathrm{value}\;\mathrm{of}\;\mathrm{word}\;i=0\\ v\left(i\right)=0,\mathrm{if}\;\mathrm{assigned}\;\mathrm{value}\;\mathrm{of}\;\mathrm{word}\;i\neq 0 \end{array}}{\mathrm{SentimentWordCount}} \end{array}$$

(5) $$\begin{array}{l} \mathrm{SentimtsgPct}=\\ \;\frac{\sum_{i}^{n}v\left(i\right)\{\begin{array}{c} v\left(i\right)=1,\mathrm{if}\;\mathrm{assigned}\;\mathrm{value}\;\mathrm{of}\;\mathrm{word}\;i=3\\ v\left(i\right)=0,\mathrm{if}\;\mathrm{assigne}\cdot d\;\mathrm{value}\;\mathrm{of}\;\mathrm{word}\;i\neq 3 \end{array}}{\mathrm{SentimentWordCount}} \end{array}$$

“ i ” is word i, “v” is value.

“ SentimentWordCount ” is the cumulative proportion of n positive emotion words.

“ SentimentNegativePct ” is the cumulative proportion of n negative emotion words.

“ SentimentNeutralPct ” is the cumulative proportion of n neutral emotion words.

“ SentimentPosNegPct ” is the cumulative proportion of n both positive and negative emotion words.

Note: We adapted Lyu’s (11) formula (which only applies to the 2008 version) to apply to the 2021 version of the Dalian University of Technology Information Retrieval Laboratory’s Chinese Affective Vocabulary Ontology Database.

#### 2.6.2 Step 2 fine-grained sentiment analysis

Furthermore, the basic sentiment polarity analysis was transcended by delving into a detailed classification of emotions that Weibo users convey. Utilizing the emotion classification framework developed by the Information Retrieval Research Laboratory at Dalian University of Technology, emotions were categorized into seven primary domains: “happiness,” “good,” “anger,” “sadness,” “fear,” “disgust,” and “surprise.” Each of these primary categories was further divided into 1–5 subcategories. For instance, the “good” emotion category was subdivided into “respect,” “praise,” “trust,” “liking,” and “wishing.”

The subtleties and shifts in public sentiment toward CCRCs were revealed by applying fine-grained sentiment analysis to Weibo texts. For example, within the broad “good” sentiment category, specific expressions of sentiment toward CCRCs can be identified. A higher frequency of “praise” suggests that CCRCs are perceived positively in services, facilities, innovation, social responsibility, and professional competence. In contrast, expressions categorized as “wish” indicate that the Weibo public desires continuous improvement in CCRCs, hoping for enhanced services and life experiences for older adults. Applying fine-grained sentiment analysis thus provided a comprehensive and precise understanding of public sentiment toward CCRCs on Weibo. Table 2 presents the categories, subcategories, and examples of the emotion lexicon based on the theory from the Information Retrieval Research Laboratory at Dalian University of Technology.

**Table 2:** Selected samples of emotion lexicon from the Dalian University of Technology Information Retrieval Research Laboratory’s Theory.

	Category	Subcategory	Sample
1	Happiness	Happy (PA)	Joyful, delighted, beaming, overjoyed
		Peaceful (PE)	Secure, relieved, reassured, guiltless
2	Good	Respectful (PD)	Respectful, revered, deferential, awestruck
		Praise (PH)	Handsome, excellent, reasonable, pragmatic
		Belief (PG)	Trusted, relied upon, dependable, unquestionable
		Adoration (PB)	Admiring, cherished, love at first sight, fond of
		Wish (PK)	Yearning, blessed, longevity and happiness, infinite life
3	Anger	Anger (NA)	Angry, infuriated, seething with rage, fuming
4	Sadness	Sadness (NB)	Sorrowful, miserable, heartrending, grief-stricken
		Disappointment (NJ)	Regretful, hopeless, disheartened, discouraged
		Remorse (NH)	Guilty, remorseful, self-reproachful, with a guilty conscience
		Thoughtful (PF)	Missing, lovesick, deeply attached, constantly in thought
5	Fear	Anxiety (NI)	Anxious, nervous, at a loss, flustered
		Fear (NC)	Timid, frightened, scared, trembling with fear
		Shame (NG)	Shy, bashful, blushing, feeling embarrassed
6	Disgust	Boredom(NE)	Frustrated, irritable, agitated, creating problems for oneself
		Hateful (ND)	Disgusted, shameful, loathed, abhorred
		Condemning (NN)	Mechanical, vain, disorganized, heartless and ruthless
		Jealous (NK)	Jealous, envious, green-eyed monster, envious of others
		Skeptical (NL)	Suspicious, mistrustful, skeptical, doubtful
7	Surprise	Surprise (PC)	Strange, miraculous, amazed, stunned

#### 2.6.3 Step 3 key emotional vocabulary analysis

Based on the previous fine-grained sentiment analysis, essential emotional vocabularies for each emotion categorization were retrieved via the TF_IDF algorithm (23). TF_IDF is the Inverse Document Frequency (IDF) of word frequency, typically used to determine the weighting of text words. It is an important measurement in the field of data mining and retrieval. Term Frequency (TF) denotes the number of times a word appears in a text. TF (i, d) refers to instances of i within the document d. IDF is an index that gauges how significant a word is. The IDF formula can be found in Equation 6, and the TF_IDF formula can be located in Equations 7, 8.


(6) $$IDF\left(i\right)=\log\left(\frac{\left|D\right|}{DF\left(i\right)+1}\right)$$

(7) $$TF\_IDF\left(i\right)=TF\left(i,d\right)\times IDF\left(i\right)$$

(8) $$TF\_IDF\left(i\right)=\sum_{d=1}^{\left|D\right|}TF\left(i,d\right)\times IDF\left(i\right)$$

|D| is the total number of documents.

$DF\left(i\right)$ is the number of documents in which the term i appears. $DF\left(i\right)+1$ is used so the denominator is never 0.

$IDF\left(i\right)$ is the IDF value of term i .

## 3 Results

Over nearly 10 years leading to the end of 2022, 9,854,050 posts were initially acquired, adopting the Weibo API and Weibo Crawler. From these, 1,027,295 posts on CCRCs were pre-processed via text denoising, stop word elimination, and word segmentation, leading to the extraction of 2,568,215 sentiment words.

### 3.1 Sentiment tendency analysis

As shown in Table 3, the text of the Weibo CCRCs includes 1,574,224 positive words, 216,191 unfavorable words, 774,182 neutral words, and 3,618 negative and positive words. The total value of sentiments was 1,358,033, which is greater than 0. Therefore, the tone of the CCRCs’ comments was overwhelmingly positive.

**Table 3:** Sentiment value of Weibo CCRCs text.

Sentiment	Frequency	Sentiment value
Positive	1,574,224	1,574,224
Negative	216,191	−216,191
Neutral	774,182	0
Both Negative and Positive	3,618	0
Total	2,568,215	1,358,033

### 3.2 Fine-grained sentiment analysis

**Figure 3:**
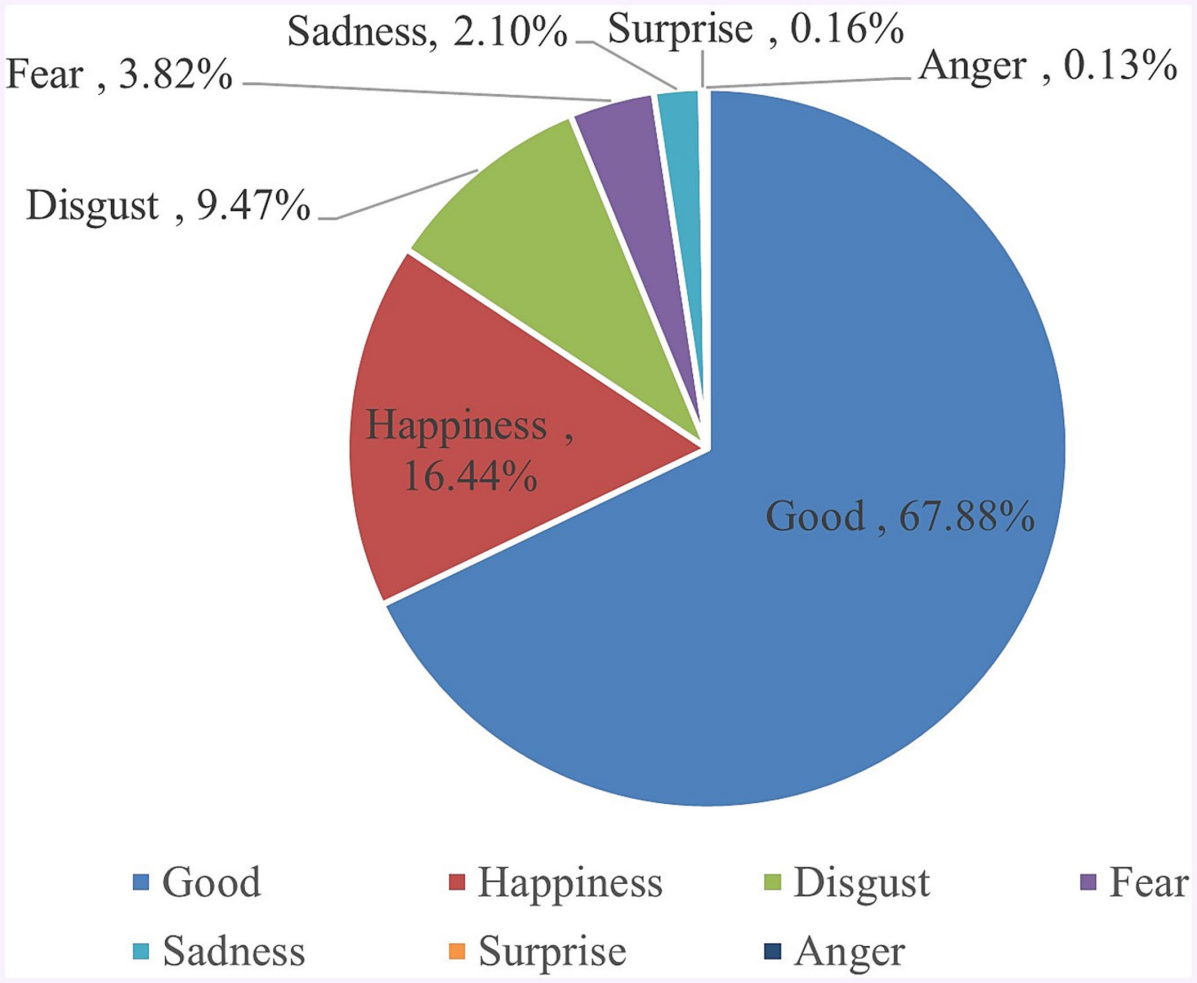
The percentage of each sentiment category.

According to the emotional vocabularies in the constructed emotion lexicon, the qualified vocabularies in the CCRCs posts were extracted. After identifying 2,568,215 emotional vocabulary words, each word was classified into emotional sub-categories, and each type of emotion’s emotional value (word frequency) was calculated. Figure 3 shows the percentage of each sentiment category, and Table 4 shows the proportion of sub-categories. It shows that 4 of 21 sub-emotional vocabulary categories account for 77.6% of the total word frequency, including 50.65% (1,300,903) from “Praise (PH),” 11.67% (299702) from “Happy (PA),” 8.65% (222162) from “Belief (PG),” 6.63% (170277) from “Condemning (NN),” higher than 5% each.

**Table 4:** The percentage of each sentiment sub-category.

Category	Sub-category	Word frequency	Proportion
Good	**Praise (PH)**	1,300,903	**50.65%**
	**Belief (PG)**	222,162	**8.65%**
	Adoration (PB)	98,288	3.83%
	Respectful (PD)	95,412	3.72%
	Wish (PK)	26,531	1.03%
Happiness	**Happy (PA)**	299,702	**11.67%**
	Peaceful (PE)	122,331	4.76%
Disgust	**Condemning (NN)**	170,277	**6.63%**
	Boredom (NE)	31,231	1.22%
	Hateful (ND)	29,071	1.13%
	Skeptical (NL)	12,540	0.49%
	Jealous (NK)	34	0.00%
Fear	Anxiety (NI)	52,912	2.06%
	Fear (NC)	44,427	1.73%
	Shame (NG)	754	0.03%
Sadness	Sadness (NB)	28,070	1.09%
	Disappointment (NJ)	9,780	0.38%
	Remorse (NH)	2019	0.08%
	Thoughtful (PF)	14,143	0.55%
Surprise	Surprise (PC)	4,231	0.16%
Anger	Anger (NA)	3,397	0.13%
		2,568,215	100%

### 3.3 TF_IDF analyze

The findings of the fine-grained sentiment analysis informed that the vast majority (77.6%) of the emotionally charged vocabulary belongs to the four sub-categories of “praise,” “happy,” “belief,” and “condemning.” Therefore, through the TF_IDF weighting algorithm, the top 5 emotional words with practical meanings for these four sub-categories were extracted. The key emotional vocabulary is shown in Table 5.

**Table 5:** Key emotion vocabulary of praise, happy, belief and condemning.

Sentiment sub-category	Vocabulary	Frequency	TF_IDF
Praise	Development	123,577	204769.65
	Health	103,239	192749.96
	Construction	101,644	184196.45
	Wisdom	36,198	100557.72
	Comprehensive	29,133	74357.16
Happy	Carry out	64,067	126921.66
	Happiness	20,276	61755.73
	Spirit	14,897	47280.23
	Enjoyment	14,677	46636.42
	Satisfaction	14,691	45206.34
Belief	Promotion	47,959	111247.30
	Guarantee	35,667	89648.070
	Support	25,381	72012.80
	Demonstration	13,069	46076.83
	People’s Government	5,394	23261.07
Condemning	Illegal	17,523	67464.15
	Fraud	13,417	52481.23
	Fever	9,237	38345.58
	Hidden danger	7,717	30694.34
	Serious/Severe	5,113	21002.33

In the comprehensive assessment of emotion vocabulary, it is evident that certain terms manifest prominently both in frequency and their TF_IDF values. Within the “praise” category, “development” leads with a frequency of 123,577 and an associated high TF_IDF score. Similarly, “carry out” dominates in the “happy” realm, while “promotion” is preeminent in expressing “belief.” Lastly, within the “condemning” sentiments, the word “illegal” becomes particularly noteworthy, achieving a frequency of 17,523 coupled with a TF_IDF measure of 67,464.15. Collectively, these terms represent their respective sentiment categories and provide a concentrated insight into the prevailing emotions in the dataset. It is worth noting that the vocabulary ranked fifth through TF_IDF in the “belief” sentiment subcategory is “must,” with a frequency of 5,567 and a TF_IDF score of 30976.66. However, it was excluded from the current analysis due to its lack of precise meaning and practical relevance.

## 4 Discussion

Analyzing Weibo comments provided insights into the prevailing public sentiment toward CCRCs. Through a sentimental analytical approach, most users were found to hold positive views toward CCRCs, though there were concerns and criticisms, as discussed in Section 4.1. Additionally, key emotional sub-categories were identified through fine-grained sentiment analysis, including “praise,” “happy,” “belief,” and “condemning.” The top five vocabulary words in each sentiment subcategory were analyzed using TF_IDF analysis and frequency statistics to facilitate thematic research, which revealed complex public perceptions, elaborated upon in Sections 4.2 to 4.5.

### 4.1 Public sentiment and perception on CCRCs

The sentiment analysis findings indicate that the texts of Weibo users related to CCRCs have a propensity toward positive sentiment. The emergence of CCRCs as a form of senior care was met with positive emotional responses on social media. Of those above, the proportion of positive emotional expression was 61.30%, while that of negative emotional expression was 8.42%. The shift in attention toward CCRCs signifies a heightened recognition of their tangible benefits and the critical requirement for institutionalized care for older people. The conventional care system, which has been dominant for a considerable period, is no longer capable of meeting the expectations of individuals. The emotional cues conveyed by these individuals signify a demand for progress, prospects, and advantages of advanced senior care service (24). When individuals communicate their “praise” and “happy,” it serves as an indication to demand increased focus and articulate persistent anticipations for more varied and advanced offerings from developers (65). Although Japan and Singapore, similar to China, are influenced by Confucian culture, their public acceptance and awareness of CCRCs vary significantly. In Japan, the public depends heavily on home-based services under long-term care insurance (25), while Singapore’s land scarcity has led to a preference for day-care centers over the CCRCs model (26).

The demands of older adults have been observed to increase in tandem with the advancement of livelihoods and progressive thinking, in addition to the Chinese government’s blueprint (27). The provision of older adult care services has witnessed a shift in the primary provider from the government to the market (4). This transition has reduced government spending while enhancing the quality of services offered to the older population. This can be attributed to the intensification of market competition. Upon examining the original Weibo post, it was discovered that individuals commonly commended CCRCs for their superior service and facility standards, the ease of access to healthcare and nursing services, the pleasant and secure living conditions, and the dynamic and engaging nature of community life (68).

Nonetheless, it is noteworthy that individuals also harbor adverse sentiments toward CCRCs. CCRCs are a relatively new concept and lack sufficient public awareness, leading to concerns over potentially fraudulent activities (78). Developers have been found to engage in illicit fundraising activities through various models such as “sales with return,” “after-sale lease,” and “agreed buyback” under the pretext of investing in CCRCs, thereby unlawfully absorbing public funds. Additionally, the inhabitants of CCRCs are typically older individuals with comparatively delicate physical and health statuses, necessitating heightened security measures, particularly in handling and resolving crises such as pandemics (28, 69).

Historically, there has been a stereotype regarding China’s societal perceptions of care for older adults, which posits that customary (29), locally established home-based care is the primary means of providing such care. However, the Chinese public’s perception of CCRCs has experienced a positive shift, as seen by the public endorsement on social media. The public has acknowledged that both the government and market possess the obligation and capability to withstand the burden of providing care for older people in their homes, as well as enhancing the quality of service in CCRCs and taking measures to combat unlawful and deceitful practices (63, 78). However, it is worth noting that China, one of the states with tight Internet control, has actively adopted soft propaganda to guide public opinion. Weibo is a case in point. Instead of completely censoring unfavorable information, the Chinese government allows the coexistence of both critical and pro-regime voices online. Where citizens can view or even discuss controversial issues. Some sharp comments may be subject to regulation, deletion, or blocking. Therefore, although a generally positive public attitude is indicated, it is important to note that some extremely negative comments may not have been captured.

### 4.2 Public awareness of the living environment in CCRCs

In the “praise” sentiment sub-category, the term that has the greatest frequency and highest TF_IDF weight value in the comments is “development.” Upon examining the source material, individuals are primarily preoccupied with ascertaining whether CCRCs possess the capacity to adequately fulfill the requirements of older people regarding matters such as health, housing, socialization, cultural engagement, and recreational activities. The establishment and healthy development of the CCRCs market should be actively supported by all social actors, including the government and businesses (30, 72).

Moreover, some are concerned about the “health” services provided by CCRCs, which go beyond routine health counseling and care to include first aid procedures and even the “green channel” that works with top-tier hospitals (Class-A tertiary hospitals in China) (31). Implementing ongoing construction is a crucial factor in ensuring quality and service progression within CCRCs (32). Furthermore, Weibo comments frequently contain phrases such as “building high-quality and high-standard CCRCs.” The construction encompasses various elements such as residential structures, communal amenities, verdant surroundings, and other related features (33). Additionally, the sector of CCRCs has also seen a rise in interest in smart facilities, management, and information services, which offer individualized, accurate, and varied senior care support for older people (34).

Finally, the concept of “comprehensive” life support and the attainment of “comprehensive” well-being for older adults is frequently referenced in discourse, encompassing various dimensions such as physical health, mental health, social health, economic status, and social participation (35). Related representative comments around the top five keywords of “Praise” as shown in Table 6.

**Table 6:** Public awareness of the living conditions in CCRCs cases.

“For older people, health and happiness are the most important things, and CCRCs should prioritize their health and happiness while also developing more health and entertainment programs suitable for them.”
“Oh, our main concern about health is whether CCRCs can arrange for someone to see a doctor at a tertiary hospital.” Serious illnesses are difficult to treat in the community.”
“CCRCs are a blessing for older people. I hope they can construct truly high-quality and high-standard CCRCs so that our children can feel secure.”
“I visited the smart construction of CCRCs and was really amazed! Older people can also enjoy the convenience and speed brought by smart technology. Truly, technology changes lives!”
“People nowadays have an open mind. If you have the means, moving into high-end retirement communities is not a bad idea. They are quite comprehensive and contain everything. It’s wonderful to spend every day with peers who are content. Inside, there is also exceptional care, with one young girl on each side of the older adult when they leave.”

The priorities of the Chinese public concerning CCRCs diverge from global viewpoints. In China, the perception of CCRCs’ living environments of older residents has traditionally been oriented toward tangible aspects such as the quality of housing, the completeness of facilities, and the accessibility of medical services (66). In contrast, public awareness in the Netherlands is more contemporary and comprehensive, emphasizing autonomy alongside social and cultural participation (36). The Humanitas Apartments for Life model suggests that overprovision of care may be more harmful than its insufficiency, presenting a marked contrast to China’s healthcare-centric model. Currently, in Chinese CCRCs, while high-quality physical living environments are often seen as paramount, developers need to proactively foresee and address residents’ future demands, which are likely to extend beyond physical aspects to encompass holistic and psychosocial dimensions of the living environment (62).

### 4.3 Carrying out activities is an important way to create a happy community atmosphere

Comments conveying a “happy” sentiment prominently featured words such as “carry out,” “happiness,” “spirit,” “enjoyment,” and “satisfaction,” which ranked both in frequency and weighted TF-IDF scores highly. CCRCs are thought to offer a vibrant and engaging living environment for older adults, encouraging a sense of community characterized by vitality, social interaction, creativity, and enjoyment (37, 60). This can contribute to cultivating a positive and purposeful outlook on life during the later stages of aging (38). One of the most commonly cited benefits of CCRCs is the ability to participate in activities, which is vital to engendering a sense of community. Shanghai Taikang Home has organized over 100 community events catering to older people’s entertainment, cultural, and social needs, offering them a diverse range of options (39). The comments presented in Table 7 are pertinent to the topic.

**Table 7:** Carrying out activities is an essential way to create a happy community atmosphere case.

“It is so heart-warming to see senior citizens living peacefully in CCRCs. When I’m old, I wish to reside there as well. To me, the community-organized events are the most alluring feature.”
“All of a sudden, I’m not as terrified of aging. They appear to be living pretty contentedly inside CCRCs, in my opinion.”
“I just purchased my folks a membership card. They have not been able to properly assimilate into life here, in my opinion, since they left their hometown. We have plenty of material possessions, but it’s crucial for them to have a companion who will make them happy.”
“I adore the way the activities are set up in CCRCs. The “forest oxygen bar” was something I had the pleasure of experiencing.
“With a wide range of daily social and recreational opportunities, living at CCRCs is incredibly satisfying. These communities can significantly improve the quality and interest of older people’s latter years.”

Similarly, CCRCs worldwide spare no effort to provide various activities. On Bribie Island in Australia, retirement villages offer a diverse and joyful atmosphere characterized by various amenities and activities promoting social interaction and cultural engagement. Popular activities include coffee socials and encouragement to participate in off-site community events (40). In Japan, while many older adults prefer aging in place, some residing in care facilities describe them as idyllic temples of long-term care due to their “precision care” philosophy. In addition to such common cultural activities as tea ceremonies, flower arranging, and calligraphy, uniquely Japanese activities such as hot springs are also organized (41). In Canada, CCRCs typically offer extensive outdoor activities and natural environments, such as community gardens, trails, and parks, which promote physical health of older adults and provide spaces for relaxation and socialization (42). These global practices highlight the importance of integrating activities and community engagement to enhance the well-being of older adults while preserving local customs and promoting regional characteristics. Chinese CCRCs developers can draw insights from these cases to create localized activities, such as encouraging outdoor activities to maintain independence (64).

### 4.4 Belief in the government’s involvement is key to people’s faith in CCRCs

Most of those Weibo posts expressing “belief” talked of how the government controls and encourages participation in establishing CCRCs. Relevant remarks reveal that the public believes the government will have a significant supervisory role in developing, maintaining, and managing CCRCs and that their participation will increase the assurances people have when deciding to settle in one (5). As a result, the government played a significant role in encouraging and supporting the growth of CCRCs in China, particularly in the beginning. Additionally, according to the Weibo post, the government supports the steady growth of CCRCs by implementing such policy tools as pilot and demonstration communities, which are similar to those used by many developed nations, including the “Life Apartment” in the Netherlands (43) and the “The Town of Soyo-Cho” in Japan (44). Related remarks are shown in Table 8.

**Table 8:** Belief in the government’s involvement is the key factor in people’s faith in CCRCs case.

“This industry can only be pushed effectively with the help of government policy incentives. It would be challenging to put CCRCs into practice without supportive policies.”
“I have faith in our government as a Chinese person. It is advisable to visit large institutions because they are more dependable and are backed by the government.”
“The government is supporting this and it is now popular. It’s got a bright future.”
“Demonstration projects are now prevalent everywhere, and everything is done by trial and error. Nobody is aware of the superior model. Anyway, let us thoroughly investigate. China currently has a serious problem with elder care.”
“I’ve witnessed the government’s noble intentions. I hope the government can keep advancing this cause and give older people more security and benefits as they age.”

Internationally, even within privately dominated systems, government support and regulation are critical for building trust and ensuring the success of CCRCs. For example, in the United States, where CCRCs originated, the government has significant influence over their operation through stringent regulatory standards and tax incentives. Public health programs such as Medicare and Medicaid also provide nursing support and financial assistance to the U.S. older adults in CCRCs (45). Therefore, in China and globally, governmental involvement is viewed as a key factor in ensuring the success of CCRCs and encouraging public trust.

### 4.5 Fear of encountering fraud and concerns over health and safety are the top causes of condemnation

The “condemning” sentiment sub-category highlights concerns over CCRCs due to worries of involvement in “illegal and fraudulent” situations. Fraud against older people has become a pressing social issue globally, with older adults being particularly vulnerable and frequently targeted (46). In China, older adults face a 10.87% probability of contact fraud and a 16.9% chance of non-contact fraud, both significantly higher than the 5.6% fraud rate experienced by their counterparts in Western countries (47). Several factors contribute to this heightened vulnerability, including cognitive decline, changes in emotional regulation, excessive trust, risk-taking behaviors, psychological weaknesses, social isolation, and limited access to fraud prevention information (46). Criminals often exploit these vulnerabilities, gaining the trust of older adults and making them easy targets for deception. Once defrauded, older people not only experience financial losses but also suffer significant psychological harm.

The growing popularity of CCRCs has given rise to a common fraudulent scheme in the care sector, where criminals attract public funds by promising high returns on investments in retirement apartments (48). These operations often lack genuine real estate transactions or use them as a front for illegal fundraising activities. Fraudsters exploit the pretense of investing in retirement properties, using tactics such as buy-back sales and after-sales leasebacks to amass funds illicitly.

In response to these growing concerns, the Chinese government issued a *“Risk Alert on Illegal Fundraising in the Older Adult Care Sector,”* emphasizing that the high-interest rates promised by such schemes are typically sustained by the payments of older adults, effectively turning these operations into Ponzi schemes. These entities generally lack sustainable operations, and when their financial structures collapse, both the promised returns and the principal investment are unlikely to be recovered due to poor regulatory controls (75). To combat these issues, the Ministry of Justice, through the 2017 *“Notice on Five Prohibitions in Notarial Practice,”* prohibited notarizations for unverifiable identities or guarantees, while the CBIRC has leveraged the *“National Illegal Fundraising Monitoring and Early Warning System (2020–2022)”* to intensify oversight of housing-for-pension scams and high-return investments. Given these risks, older people should exercise caution when confronted with offers that exceed typical market returns, as they are often fraudulent (49, 58). Moreover, emotional support from families plays a crucial role in protecting older adults from fraud that preys on their need for affection (50).

Additionally, “fever” is a common concern of people. The physical well-being of older adults is of utmost importance in CCRCs, so institutions should take appropriate precautions to ensure their health and security (51). The novel coronavirus outbreak has presented significant challenges for CCRCs worldwide in preventing epidemics (52). The media indicated that due to poor management or a lack of appropriate epidemic prevention measures, various domestic and foreign institutions may have contributed to a significant number of older persons becoming sick and experiencing fever symptoms. In American CCRCs, studies have highlighted the pandemic’s devastating impact, with operators feeling unprepared and exhausted. In Australia, most CCRC residents viewed lockdown as a reasonable public health measure and acknowledged managers’ efforts under tough conditions, yet still expressed disappointment and stress over health (53). In China, while stringent measures such as comprehensive lockdowns effectively contained the virus, they also resulted in heightened resident dissatisfaction and isolation, contradicting their initial expectations of community life (54). Thus, CCRCs globally need to develop more effective health measures to ensure residents’ safety and well-being while balancing their psychological welfare and social connections. Moreover, numerous comments brought up additional possible issues with CCRCs that were not considered adequately, such as food, fire, and medical safety (55, 70). Related remarks are shown in Table 9.

**Table 9:** Fear of encountering fraud and concerns about health and safety rank as the top causes of condemning case.

“There are still many who claim to be providing care for older people but are just selling houses. It’s a classic case of false advertising, and it happens everywhere.”
“The idea of catching a contagious disease in a place where a lot of older people gather is pretty scary…why would anyone still want to live in a CCRCs?”
“During the pandemic, all the nursing homes were on lockdown and family members could not visit. It’s understandable that they were being cautious, but it was also inconvenient. We just have to try to understand each other’s perspectives.”
“Let us crack down on scams targeting older adults! There are way too many shady practices going on in health clubs and fitness centers.”
“When there are food safety issues in the cafeteria, who’s to blame? CCRCs management must be more vigilant about this.”

Anyone can engage in online discussion forums in the digital era and use their voice to pressure authorities. People have high expectations of the government in such an Internet age where information openness is desperately needed since it is the last bastion of security and longevity. The public also expressed hope that the government would bolster its efforts to combat illicit fraud and control infectious diseases, such as pneumonia, at the same time. Condemning remarks are not serious yet, but they show to some extent that the Chinese government is slow to respond to the public and lacks a reliable system for handling public opinion. Therefore, the ongoing senior care fraud occurrences and the widespread CCRC infection have caused individuals to become unsatisfied with and wary of CCRCs. Government management has undergone significant changes in the information era (56). Network governance is an important strategy for raising management effectiveness. The government could change the conventional practice of giving directives through formal channels and instead aggressively address public concerns and disseminate health and safety information on various venues (57).

### 4.6 Limitations and future research

The study also has certain limitations. It primarily relies on data from a single social media platform. Discussions about CCRCs are likely occurring across various online forums, WeChat groups, and other social media platforms. While Weibo has a substantial user base in China, it is premature to assume that the opinions expressed there fully capture broader public sentiment. Future research would benefit from integrating data from diverse social media platforms and tailoring data weighting to reflect the demographic characteristics of each platform’s users, ensuring a more precise and representative measure of public sentiment. Another constraint of relying on social media data is that platforms often remove posts with extreme sentiments, potentially leading to an underrepresentation of certain emotions in our samples. To enhance the analysis, future research could integrate news reports, questionnaires, or surveys, as these may more effectively capture extreme sentiments. Additionally, the analysis is confined to online comments on social media and relies solely on text sentiment analysis, excluding other pertinent data such as the number of likes and shares. Integrating these data with sentiment analysis could offer a more comprehensive understanding of the spread and influence of social media users’ emotional attitudes and behaviors. Furthermore, given that CCRCs are still in their early stages of development in China, numerous operational and managerial challenges persist, and public opinions of them may also shift soon. Future research needs to focus on longitudinal studies to understand how the evolution of CCRC operations and management practices influences public perceptions and acceptance as these facilities develop and improve. Moreover, while this study situates the Chinese experience within the global context for comparison and contrast, a detailed analysis of the policy, cultural, and economic characteristics behind the differing performances of CCRCs in various countries remains to be further explored. Future research can address this gap to understand how these factors influence the successful implementation of CCRCs, providing more practical insights for policy and practice.

## 5 Conclusion

The escalating demand for senior care in China necessitates delegating a portion of the responsibility from conventional home care and government care to the market. CCRCs are a form of institutional senior care that has emerged as a market investment, operation, and management strategy under such a background. Despite being relatively new in China, CCRCs have attracted significant public interest, with individuals utilizing such social media platforms as Weibo to share their perspectives and opinions. Analysis of public sentiment regarding CCRCs is currently inadequate. This study addresses the deficiency mentioned above by conducting a comprehensive and systematic examination of comments related to Weibo through natural language techniques.

Based on sentiment analysis, this study presents three progressive findings. Statistics show that the prevailing attitude is positive in the context of CCRCs. Subsequently, following a fine-grained analysis, it has been determined that the primary attitudes of the populace toward CCRCs are “praise,” “belief,” “happiness,” and “condemning.” Thirdly, after TF_IDF weighted analysis, several key characteristics are identified. The living environment of CCRCs is a topic of public interest. Engaging in activities within CCRCs is critical for encouraging a positive community atmosphere, a primary motivator for residents to relocate. The involvement of government entities is a key factor in instilling trust in individuals toward CCRCs. Concerns regarding potential fraud and issues pertaining to health and safety are significant factors that contribute to the negative perception of CCRCs. Weibo posts reflect the public’s focus and emotional attitudes toward these facilities. Even though the percentage of disparaging remarks is not high, they cannot be disregarded. If the issue is not effectively addressed, it would undermine public trust and confidence in the government’s management and regulation of these facilities.

Furthermore, in contemporary times, the Internet serves as a valuable platform for individuals to articulate their viewpoints. The confluence of social media advancement and the political climate in mainland China has led to an inescapable outcome: opinion orientation is dominated by the government, owing to the country’s vast populace and the centralized government’s control over mainstream media. They emerged as a significant platform for Chinese individuals to articulate their sentiments and viewpoints and potentially engage in political activities.

In summary, our work exemplifies how social media can be used to monitor public opinion and sentiment toward emerging new phenomena. This study utilizes NLP technology to reveal the public’s favorable attitudes and unfavorable factors toward CCRCs. It also uncovers critical aspects of the community living environment, community activities and atmosphere, government participation, industry fraud, and health and safety issues. This study provides valuable insights for the government, developers, and residents, enhancing understanding and offering solid reference information for subsequent planning and decision-making regarding CCRCs, especially in addressing transitional and transformational uncertainties.

## Data Availability

The raw data supporting the conclusions of this article will be made available by the authors, without undue reservation.
