# Supplementary material for: Exploring public attitudes of continuing care retirement communities in China: a sentiment analysis of China’s social media Weibo
Source: Front Public Health. 2025 Feb 18;12:1454287. doi: 10.3389/fpubh.2024.1454287 (PMC11875845; doi:10.3389/fpubh.2024.1454287)
Supplement: Supplementary file 1 [file Data_Sheet_1.docx]

**Appendices**

**Appendix 1 CCRC dictionary**

| CCRCs dictionary |
| --- |
| CCRC, 老年社区(Laonian shequ),退休住宅(Tuixiu zhuzhai), 养生地产(Yanglao dichan), 退休社区(Tuixiu shequ), 养老住宅(Yanglao zhuzhai), 书院式养老(Shuyuanshi yanglao), 养老公寓(Yanglao gongyu), 旅居养老(Lvju yanglao),养老产业园(Yanglao chanyeyuan), 养老地产(Yanglao dichan), 养老养生度假中心(Yanglao yangsheng dujia zhongxin), 智慧养老(Zhihui yanglao), 养老综合服务社区(Yanglao fuwu zonghe shequ), 机构养老(Jigou yanglao), 持续照料退休社区(Chixu zhaoliao tuixiu shequ), 老年公寓 (Laoian gongyu), 综合养老生态社区(Zonghe yanglaqo shengtai shequ), 老年地产(Laonian dichan), 持续照顾型退休住宅(Chixu zhaoguxing tuixiu zhuzhai), 养老综合体(Yanglao zongheti), 养老学院(Yanglao xueyuan), 医养综合体(Yiyang jieheti), 大健康项目(Dajiankang xiangmu), 学院式养老(Shuyuanshi Ynaglao), 养老社区(Yanglao shequ), 康养综合体(Kangyang zongheti), 养老设施(Yanglao sheshi), 镶嵌式养老(Xiangqianshi yanglao), 医养结合(Yiyang jiehe),亲情养老住宅(Qinqing yangao zhuzhai), 康养产业(Knagyang chanye), 全龄养老社区(Quanling yanglaoshequ), 康养小镇(Kangyang xiaozhen), 养老商业地产(Shangye yanglao dichan), 康养设施(Kangyang sheshi), 互联网+养老(Hulianwang + Yanglao), 高级老人公寓(Gaoji laoren gongyu),养老机构(Yanglao jigou),养老设施和服务(Yanglao sheshi he fuwu), 旅游养老(Lvyou yanglao) |

**Appendix 2. Pseudo code for data cleaning**

| input: L, a set contains all collected posts  output: L*, the cleaned post list  L* = L  for every single post i in L do  if checkKeywords(i) == false then  L* = L* \ {i};  End if  if checkDuplicate(i) == false then  L* = L* \ {i};  End if  If checkDate(i) == false then  L* = L* \ {i};  End if  if checkRabbishandBot(i) == false then  L* = L* \ {i};  End if  End for  Return L* |
| --- |

**Appendix 3 Example of pre-processing post.**

| Original sentence | 回复@俊：绿城养老地产项目一步一景，老年大学助餐食堂康复医院配套齐全。反思这样的养老项目是否适合自己，远离城市，远离既往社交圈？目前我是不会选择，mark一下，看看多年后是否改变 |
| --- | --- |
|  | Huifu@Jun:Lvchengyanglaodichanxiangmuyibuyijing,laoniandaxuezhucanshitangkangfuyiyuanpeitaoqiquan.Fansizheyangdeyanglaoxaingmushifoushiheziji,yuanlichengshi,yuanlijiwangshejiaoquan?muqianwoshibuhuixaunze,markyixia,kankanduonianhoushifougaibian |
| Word segmentation | 回复 @ 俊：绿城 养老 地产 项目 一步 一景，老年大学 助餐 食堂 康复 医院 配套 齐全 。 反思 这样 的 养老 项目 是否 适合 自己.远离 城市.远离 既往 社交圈 ？ 目前 我 是 不会 选择. mark 一下. 看看 多年 后 是否 改变 |
|  | Huifu @ Jun: Lvcheng yanglao dichan xiangmu yibu yijing. laoniandaxue zhucan shitang kangfu yiyuan peitao qiquan . Fansi zheyang de yanglao xaingmu shifou shihe ziji. yuanli chengshi. yuanli jiwang shejiaoquan ? muqian wo shi buhui xaunze. mark yixia. Kankan duonian hou shifou gaibian |
| Data denoise | 绿城 养老 地产 项目 一步 一景 老年大学 助餐 食堂 康复 医院 配套 齐全 反思这样 的 养老 项目 是否 适合 自己 远离 城市 远离 既往 社交圈 目前 我 是 不会 选择 mark 一下 看看 多年 后 是否 改变 |
|  | Lvcheng yanglao dichan xiangmu yibu yijing laoniandaxue zhucan shitang kangfu yiyuan peitao qiquan Fansi zheyang de yanglao xaingmu shifou shihe ziji yuanli chengshi yuanli jiwang shejiaoquan muqian wo shi buhui xaunze mark yixia Kankan duonian hou shifou gaibian |
| Stop word removal | 绿城 养老 地产 项目 一步 一景 老年大学 助餐 食堂 康复 医院 配套 齐全 反思 养老 项目 是否 适合 远离 城市 远离 既往 社交圈 目前 不会 选择 mark 一下 看看 多年 后 是否 改变 |
|  | Lvcheng yanglao dichan xiangmu yibu yijing laoniandaxue zhucan shitang kangfu yiyuan peitao qiquan fansi yanglao xiangmu shifou shihe yuanli chengshi yuanli jiwang shejiaoquan muqian buhui xuanze mark yixia Kankan duonian hou shifou gaibian |
